# Predicting Persistent Left Ventricular Dysfunction After Cardiac Resynchronization Therapy Upgrade in Pacing-Induced Cardiomyopathy

**DOI:** 10.3390/jcm15218731

**Published:** 2026-11-09

**Authors:** Jakub Simka, Rudolf Praus, Jiri Jager, Martin Matatko, Monika Fialova, Eva Cermakova, Jakub Stritecky, Ludek Haman, Petr Parizek

**Affiliations:** 1 First Department of Internal Medicine Cardiology and Angiology, University Hospital Hradec Kralove, 500 05 Hradec Kralove, Czech Republic; 2 Faculty of Medicine in Hradec Kralove, Charles University, 500 03 Hradec Kralove, Czech Republic

**Keywords:** pacing-induced cardiomyopathy, cardiac resynchronization therapy, CRT upgrade

## Abstract

**Background/Objectives:** Pacing-induced cardiomyopathy (PICM) is potentially reversible after cardiac resynchronization therapy (CRT) upgrade, but severe left ventricular dysfunction persists in some patients. We evaluated predictors of persistent left ventricular ejection fraction (LVEF) ≤ 35% after CRT upgrade and long-term outcomes. **Methods:** We analysed 60 patients undergoing CRT upgrade for PICM. The primary outcome was persistent LVEF ≤ 35% at 12 months. Clinical, electrocardiographic, echocardiographic, and long-term outcomes were assessed. **Results:** At 12 months, median LVEF increased from 30% to 45% (*p* < 0.01), and 82% of patients recovered to an LVEF > 35%. Persistent severe left ventricular dysfunction was associated with lower baseline LVEF (22% vs. 30%; *p* < 0.01), greater left ventricular end-diastolic diameter (LVEDD) (65 mm vs. 58 mm; *p* = 0.03), and longer post-CRT QRS duration (154 ms vs. 140 ms; *p* = 0.02); none remained independently significant after multivariable adjustment. No patient with baseline LVEF > 25% and LVEDD < 65 mm had persistent severe LV dysfunction, compared with 46% of those with LVEF ≤ 25% and LVEDD ≥ 65 mm. In the 12-month landmark analysis, mortality through 60 months after CRT upgrade was 55% in patients with persistent severe LV dysfunction and 31% in those with LVEF recovery. **Conclusions:** CRT upgrade was associated with substantial recovery of LV systolic function in patients with PICM. Severe baseline LV dysfunction and LV dilatation were associated with a lower likelihood of recovery to an LVEF > 35%. These exploratory findings may aid risk stratification but require prospective validation.

## 1. Introduction

Right ventricular (RV) pacing alters the physiological pattern of ventricular activation, resulting in electrical and mechanical dyssynchrony [1,2,3]. In susceptible patients, chronic exposure to this abnormal activation pattern may lead to progressive left ventricular (LV) systolic dysfunction and heart failure, a condition referred to as pacing-induced cardiomyopathy (PICM) [4]. This condition may develop in up to one-quarter of chronically paced patients and is potentially reversible [5]. Restoration of ventricular synchrony by upgrading to cardiac resynchronization therapy (CRT) may result in substantial LV reverse remodelling and recovery of systolic function [6]. In a selected population of patients with PICM and severely reduced left ventricular ejection fraction (LVEF), 72% recovered to an LVEF > 35% following CRT upgrade, with most improvement occurring within the first year [7].

The high rate of LV recovery after CRT upgrade raises an important clinical question regarding the need for defibrillator backup at the time of upgrade. Correction of electromechanical dyssynchrony, QRS narrowing, improvement in LVEF, and subsequent LV reverse remodelling may reduce the risk of life-threatening ventricular arrhythmias [8,9,10]. Observational studies have reported a relatively low incidence of malignant ventricular arrhythmias after CRT upgrade in patients with PICM and have questioned the incremental benefit of adding an implantable cardioverter-defibrillator (ICD) in this setting [11,12].

Despite the high probability of reverse remodelling, a subset of patients with PICM remains with severely impaired LV systolic function after CRT upgrade. However, predictors of persistent LVEF ≤ 35% are poorly defined. We therefore sought to identify clinical, electrocardiographic, and echocardiographic predictors of persistent severe LV dysfunction following CRT upgrade in patients with PICM and to identify patients with a high likelihood of recovery above the 35% LVEF threshold.

## 2. Materials and Methods

### 2.1. Study Population

The present study is a retrospective secondary analysis of patients enrolled in the prospective Long-Term Outcomes and Predictors of Reverse Remodelling After Cardiac Resynchronization Therapy Upgrade study [13]. The original cohort included consecutive patients undergoing CRT upgrade between October 2018 and September 2020 at University Hospital Hradec Kralove, Czech Republic. For the present analysis, only patients in whom PICM was the indication for CRT upgrade were included. PICM was defined as an LVEF < 50% with an absolute decrease of ≥10 percentage points from baseline in the presence of a right ventricular pacing burden of ≥40%. This definition was based on previously published criteria, with a ≥40% RV pacing threshold selected to ensure substantial exposure to RV pacing [4,7]. Alternative primary causes of the observed decline in LV systolic function, including active myocardial ischemia, significant valvular disease, and other cardiomyopathies, were clinically excluded. All patients provided written informed consent for participation in the original study and for prospective collection and analysis of clinical data. The study protocol was approved by the Ethics Committee of University Hospital Hradec Kralove and complied with the Declaration of Helsinki.

### 2.2. Study Endpoints

Persistent severe LV systolic dysfunction was defined as an LVEF ≤ 35% at 12 months after CRT upgrade. This classification was based on LVEF at 12 months rather than on conventional CRT response criteria. The primary objective was to identify clinical, electrocardiographic, and echocardiographic predictors of persistent severe LV dysfunction and to compare patients with an LVEF ≤ 35% with those who recovered to an LVEF > 35%. Secondary endpoints included long-term all-cause mortality according to the presence or absence of persistent severe LV dysfunction and according to LV reverse remodelling status. In an additional analysis, LV reverse remodelling was defined as an absolute increase in LVEF of ≥10 percentage points at 12 months after CRT upgrade. Clinical, electrocardiographic, and echocardiographic predictors of reverse remodelling were also evaluated.

### 2.3. Data Collection and Follow-Up

Before CRT upgrade, all patients underwent clinical evaluation, device interrogation, 12-lead electrocardiography, laboratory testing, and transthoracic echocardiography. Clinical evaluation included assessment of New York Heart Association (NYHA) functional class, and device interrogation was used to quantify the RV pacing burden. Laboratory measurements included routine hematologic and biochemical parameters and N-terminal pro–B-type natriuretic peptide (NT-proBNP). Echocardiographic assessment included LVEF, cardiac chamber dimensions, including left ventricular end-diastolic diameter (LVEDD), valvular disease, and pulmonary hypertension. LVEF was measured with the biplane Simpson method when technically feasible and was estimated visually when image quality precluded reliable volumetric assessment. Pulmonary hypertension was assessed by Doppler echocardiography using peak tricuspid regurgitation velocity, estimated systolic pulmonary artery pressure, and additional echocardiographic signs, in accordance with the 2015 ESC/ERS guidelines [14]. Based on estimated systolic pulmonary artery pressure, pulmonary hypertension was classified as mild (35–<45 mmHg), moderate (45–60 mmHg), or severe (>60 mmHg). Additional clinical data, including comorbidities, previous cardiac surgery, and findings from coronary angiography, were obtained from the hospital information system. CRT upgrade procedures were performed by experienced electrophysiologists in a dedicated device implantation laboratory. The choice of CRT-P (cardiac resynchronization therapy with pacemaker) or CRT-D (cardiac resynchronization therapy with defibrillator) was based on contemporary guideline recommendations and individual clinical assessment. Post-CRT QRS duration was measured at the end of the procedure. All patients underwent clinical evaluation and device interrogation 3 months after CRT upgrade. Thereafter, patients with CRT-D were routinely followed every 6 months and those with CRT-P annually. At 12 months after CRT upgrade, all patients underwent clinical evaluation, device interrogation, 12-lead electrocardiography, laboratory testing, and repeat transthoracic echocardiography. Follow-up echocardiographic examinations were performed by a single experienced cardiologist to minimize interobserver variability. Appropriate ICD therapies were recorded at scheduled device interrogations and at unscheduled evaluations when clinically indicated. Long-term vital status was ascertained from regularly updated health insurance records.

### 2.4. Statistical Analysis

Continuous variables are presented as medians with interquartile ranges and categorical variables as counts and percentages. Normality was assessed with the Shapiro–Wilk test. Paired and between-group comparisons were performed with the Wilcoxon signed-rank and Mann–Whitney U tests, respectively; categorical variables were compared with the chi-square or Fisher’s exact test, as appropriate. Associations with persistent severe LV dysfunction were assessed by logistic regression and are reported as odds ratios with 95% confidence intervals. Five variables with *p* < 0.10 in univariable analyses were included in the multivariable model. Given the limited number of events, multivariable estimates were considered exploratory and obtained with Firth penalized logistic regression. Predictors of LV reverse remodelling were assessed by univariable logistic regression. Long-term survival according to 12-month LV function was assessed using a 12-month landmark analysis among patients with available 12-month LVEF data. Survival was analyzed with Kaplan–Meier estimates, the log-rank test, and Cox proportional-hazards models. All tests were two-sided, with *p* < 0.05 considered statistically significant.

## 3. Results

### 3.1. Study Population and Baseline Characteristics

During the study period, 65 patients underwent CRT upgrade for PICM. Five patients did not complete the 12-month follow-up assessment: three died before the scheduled visit (one from heart failure, one from lung cancer, and one from COVID-19), and two were unable to attend follow-up. The final study cohort therefore comprised 60 patients with available 12-month follow-up data. The median age was 75 (IQR 72–80) years, and 21 patients (35%) were female. Ischemic heart disease was present in 24 patients (40%), while atrial fibrillation was highly prevalent, affecting 42 patients (70%). Relevant comorbidities included arterial hypertension in 51 (85%), diabetes mellitus in 24 (40%), and chronic kidney disease stage ≥ 3 in 37 patients (62%). The median time from initial device implantation to CRT upgrade was 64 (IQR 25–99) months, with a median RV pacing burden of 99% (IQR 94–100%). Before upgrade, 55 patients (92%) had a conventional pacemaker and 5 (8%) an ICD. CRT-P was implanted in 43 patients (72%), whereas 17 (28%) received CRT-D. Baseline characteristics are summarized in Table 1.

### 3.2. Clinical and Echocardiographic Response to CRT Upgrade

At 12-month follow-up, CRT upgrade was associated with significant electrical, echocardiographic, and clinical improvement. Median QRS duration decreased from 187 (IQR 175–200) ms to 140 (IQR 134–153) ms, *p* < 0.01. This was accompanied by substantial LV reverse remodelling, with LVEF increasing from 30% (IQR 24–35%) at baseline to 45% (IQR 37–52%) after 12 months of follow-up, *p* < 0.01; and LVEDD decreasing from 58 (IQR 54–64) mm to 56 (IQR 51–61) mm, *p* < 0.01. Clinical improvement was reflected by a reduction in NT-proBNP from 2017 (IQR 978–4824) ng/L to 1220 (IQR 642–2310) ng/L, *p* < 0.01; and a significant improvement in NYHA functional class *p* < 0.01. Pulmonary hypertension also decreased significantly during follow-up (*p* < 0.01), whereas changes in mitral and tricuspid regurgitation did not reach statistical significance (*p* = 0.07 and *p* = 0.62, respectively). Detailed changes in clinical, laboratory, electrocardiographic, and echocardiographic parameters are summarized in Table 2.

### 3.3. Patients with Persistent Severe LV Dysfunction

At 12-month follow-up, 11 patients (18%) had persistent severe LV systolic dysfunction, whereas 49 patients (82%) recovered to an LVEF > 35%. Patients with persistent severe LV dysfunction had significantly lower baseline LVEF (22% [IQR 20–25%] vs. 30% [IQR 25–35%]; *p* < 0.01) and greater baseline LVEDD (65 mm [IQR 59–70 mm] vs. 58 mm [IQR 54–61 mm]; *p* = 0.03). They also had a longer post-CRT QRS duration (154 ms [IQR 139–164 ms] vs. 140 ms [IQR 132–148 ms]; *p* = 0.02), whereas the magnitude of QRS shortening was similar between groups (*p* = 0.89). Ischemic heart disease was numerically more frequent among patients with persistent severe LV dysfunction (64% vs. 35%; *p* = 0.10). No significant differences were observed in age, RV pacing burden, time to CRT upgrade, atrial fibrillation, other major comorbidities, or baseline heart failure therapy (Table 3).

In univariable logistic regression, higher baseline LVEF was associated with lower odds of persistent severe LV dysfunction (OR 0.46 per 5-percentage-point increase, 95% CI 0.26–0.81; *p* = 0.01), whereas greater baseline LVEDD (OR 3.73 per 10-mm increase, 95% CI 1.34–10.41; *p* = 0.01) and longer post-CRT QRS duration (OR 1.81 per 10-ms increase, 95% CI 1.11–2.96; *p* = 0.02) were associated with higher odds of the primary outcome. Ischemic heart disease was not significantly associated with persistent severe LV dysfunction (OR 3.29, 95% CI 0.84–12.86; *p* = 0.09). Arterial hypertension was inversely associated with the outcome in univariable analysis (OR 0.20, 95% CI 0.04–0.93; *p* = 0.04). Variables with a univariable *p* < 0.10 were entered into the multivariable model. In multivariable analysis, none of the variables remained independently associated with persistent LVEF ≤ 35% after adjustment. Baseline LVEF showed the strongest residual association, although this did not reach statistical significance (adjusted OR 0.63 per 5-percentage-point increase, 95% CI 0.32–1.24; *p* = 0.18). Detailed univariable and multivariable analyses are presented in Table 3. Post-hoc stratification according to baseline LVEF and LVEDD showed a stepwise increase in the prevalence of persistent severe LV dysfunction (Table 4). None of the 27 patients with baseline LVEF > 25% and LVEDD < 65 mm had persistent severe LV dysfunction at 12 months. The prevalence increased to 25% (1 of 4 patients) among those with an LVEF > 25% and an LVEDD ≥ 65 mm and to 28% (5 of 18 patients) among those with an LVEF ≤ 25% and an LVEDD < 65 mm. The highest prevalence, 46% (5 of 11 patients), was observed among patients with both an LVEF ≤ 25% and an LVEDD ≥ 65 mm (*p* < 0.01).

### 3.4. Long-Term Outcomes

**Figure 1:**
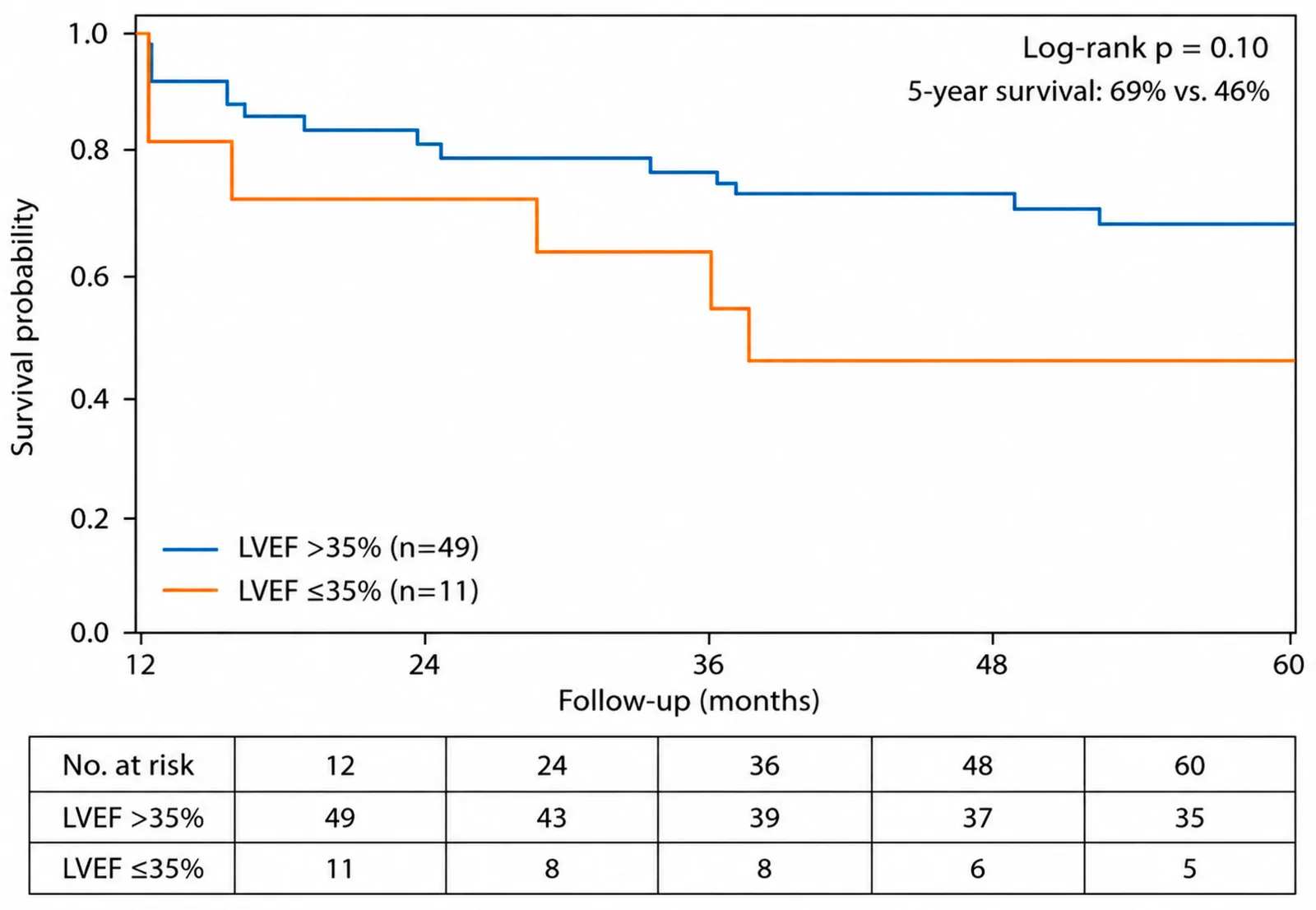
Kaplan–Meier estimates of survival in a 12-month landmark analysis according to left ventricular systolic function at 12 months after CRT upgrade. Survival is shown for patients with recovery of LVEF to >35% and those with persistent severe LV systolic dysfunction (LVEF ≤ 35%). The numbers of patients at risk are shown below the graph. The between-group difference was assessed with the log-rank test. During follow-up, one patient in each group received appropriate ICD therapy.

In the 12-month landmark analysis, 6 of 11 patients (55%) with persistent severe LV dysfunction and 15 of 49 patients (31%) with recovery of LVEF to >35% died during follow-up through 60 months after CRT upgrade. Among the 21 patients who died, 4 deaths were cardiovascular and 4 were non-cardiovascular; the cause of death remained unknown in the remaining 13 patients. The estimated 5-year survival according to Kaplan–Meier analysis was 46% and 69%, respectively. Mortality was numerically higher among patients with severe persistent LV dysfunction, however, the survival difference between the groups was not significant and estimates were imprecise (HR 2.17; 95% CI 0.84–5.60; log-rank *p* = 0.10) (Figure 1). Appropriate ICD therapy occurred in only two patients in the entire cohort, one of whom had persistent severe LV dysfunction. Among patients with LV reverse remodelling, 15 of 47 (32%) died during 60 months of follow-up, as compared with 6 of 13 patients (46%) without reverse remodelling. Survival did not differ significantly between the groups (HR 0.85, 95% CI 0.34–2.13; log-rank *p* = 0.36).

### 3.5. Reverse Remodelling

Reverse remodelling, defined as an absolute increase in LVEF of ≥10 percentage points, occurred in 47 of 60 patients (78%). No significant differences in baseline clinical, electrocardiographic, or echocardiographic characteristics were observed between patients with and without reverse remodelling. Patients without reverse remodelling showed numerically greater baseline LVEDD (61 mm [IQR 57–65 mm] vs. 58 mm [IQR 54–61 mm]; *p* = 0.11) and longer post-CRT QRS duration (146 ms [IQR 138–158 ms] vs. 140 ms [132–150 ms]; *p* = 0.14), although neither difference reached statistical significance (Table 5). The prevalence of ischemic heart disease, atrial fibrillation, diabetes, chronic kidney disease, and baseline heart failure therapy was comparable between groups. Consistently, univariable logistic regression identified no significant predictors of LV reverse remodelling. Greater baseline LVEDD showed only a nonsignificant trend toward a lower likelihood of reverse remodelling (OR for non-response 2.18 per 10-mm increase, 95% CI 0.92–5.12; *p* = 0.08).

**Table 5:** Baseline clinical, electrocardiographic, and echocardiographic characteristics according to left ventricular reverse remodelling after CRT upgrade. Left ventricular reverse remodelling was defined as an absolute increase in LVEF of ≥10 percentage points at 12 months after CRT upgrade.

Variable	LVEF Improvement ≥ 10% (*n* = 47)	LVEF Improvement < 10% (*n* = 13)	*p*
Age—years, median (IQR)	76 (74–80)	74 (67–79)	0.18
Male sex—*n* (%)	29 (62)	10 (77)	0.51
Time to CRT upgrade—months, median (IQR)	59 (18–98)	89 (35–99)	0.26
RV pacing—%, median (IQR)	99 (95–100)	97 (92–100)	0.42
BMI—kg/m^2^, median (IQR)	29 (27–31)	27 (26–34)	0.79
NT-proBNP—ng/L, median (IQR)	2113 (1175–4591)	1821 (536–6935)	0.82
Post-CRT QRS complex—ms, median (IQR)	140 (132–150)	146 (138–158)	0.14
QRS complex change—ms, median (IQR)	46 (36–56)	42 (36–54)	0.96
Baseline LVEF—%, median (IQR)	30 (24–35)	30 (25–35)	0.88
Baseline LVEDD—mm, median (IQR)	58 (54–61)	61 (57–65)	0.11
Ischemic heart disease—*n* (%)	18 (38)	6 (46)	0.75
Arterial hypertension—*n* (%)	41 (87)	10 (77)	0.39
Atrial fibrillation—*n* (%)	33 (70)	9 (69)	1.00
Valvular disease—*n* (%)	12 (26)	6 (46)	0.18
COPD—*n* (%)	4 (9)	2 (15)	0.60
Diabetes mellitus—*n* (%)	17 (36)	7 (54)	0.34
Anaemia—*n* (%)	19 (40)	4 (31)	0.75
Chronic kidney disease—*n* (%)	31 (66)	6 (46)	0.22
ACEi/ARB/ARNI—*n* (%)	35 (75)	9 (69)	0.73
MRA—*n* (%)	20 (43)	5 (39)	1.00
Beta-blocker—*n* (%)	39 (83)	11 (85)	1.00

Abbreviations: LVEF, left ventricular ejection fraction; IQR, interquartile range; BMI, body mass index; NT-proBNP, N-terminal pro–B-type natriuretic peptide; LVEDD, left ventricular end-diastolic diameter; CRT, cardiac resynchronization therapy; COPD, chronic obstructive pulmonary disease; ACEi, angiotensin converting enzyme inhibitor; ARB, angiotensin receptor blocker; ARNI, angiotensin receptor and neprilysin; MRA, mineralocorticoid receptor antagonist.

### 3.6. Complications

During follow-up, one patient underwent surgical revision for a device-pocket hematoma. Three patients (5%) underwent system extraction for infection, including two with cardiac device-related infective endocarditis, and one patient underwent replacement of the left ventricular lead because of lead dysfunction.

## 4. Discussion

The principal finding of this study is the substantial recovery of LV systolic function after CRT upgrade for PICM. At 1 year, 82% of patients had recovered to an LVEF > 35%, with a median increase in LVEF from 30% to 45%. This improvement was accompanied by LV reverse remodelling, QRS narrowing, lower NT-proBNP levels, and improved NYHA functional class. These findings support the concept that PICM is a potentially reversible form of LV dysfunction when pacing-related ventricular dyssynchrony is corrected. Our results are consistent with those of Khurshid et al., who reported an increase in mean LVEF from 29% to 45% after CRT upgrade, with 72% of patients with severe pacing-induced cardiomyopathy recovering to an LVEF > 35% [7]. Similarly, other contemporary cohorts have demonstrated substantial improvement in LV function and functional status following CRT upgrade in patients with PICM [8,11,15,16,17,18,19]. Beyond reverse remodelling, the clinical benefit of CRT upgrade has been demonstrated in the randomized BUDAPEST-CRT Upgrade trial. Among symptomatic patients with reduced LVEF, a high right ventricular pacing burden, and a wide paced QRS complex, upgrade to CRT-D, as compared with ICD therapy alone, reduced the risk of the composite outcome of death, hospitalization for heart failure, or absence of substantial LV reverse remodelling at 12 months [20]. Importantly, the benefit of CRT upgrade was consistent across patients with sinus rhythm and those with atrial fibrillation [21]. Conduction system pacing, particularly left bundle branch area pacing (LBBAP), has emerged as an alternative approach to CRT. Observational studies suggest that LBBAP can improve LV function in patients with PICM, with outcomes comparable to biventricular pacing and, in some studies, greater LV reverse remodelling [22,23,24,25,26]. However, randomized evidence comparing these strategies specifically in patients undergoing CRT upgrade remains limited.

Despite the overall favorable response, approximately one in five patients had persistent severe LV dysfunction at 1 year. These patients had lower baseline LVEF, greater LVEDD, and longer post-CRT QRS duration. Although these variables were associated with persistent LV dysfunction in univariable analyses, none remained independently significant after multivariable adjustment, likely owing to the limited number of events and the interrelation among markers of advanced LV remodelling. The combination of baseline LVEF and LV dilatation provided additional prognostic information. No patient with an LVEF > 25% and an LVEDD < 65 mm had persistent severe LV dysfunction, whereas this outcome occurred in 46% of patients with both an LVEF ≤ 25% and an LVEDD ≥ 65 mm. These findings suggest that baseline LV characteristics may help identify patients most likely to recover after CRT upgrade. However, given the small subgroup sizes, the proposed thresholds should be considered exploratory and require external validation. Persistent severe LV dysfunction was associated with numerically higher long-term mortality (55% vs. 31%), although the difference was not significant. Notably, appropriate ICD therapy occurred in only two of the 17 CRT-D recipients during follow-up. Loboda et al. identified severe LV systolic dysfunction with LVEF < 20% before CRT upgrade as a predictor of mortality, and an LVEF ≤ 25% has been associated with sustained ventricular arrhythmias in previous pacemaker recipients without a history of ventricular arrhythmia [12]. Similarly, Tayal et al. identified an LVEF < 24% as a negative predictor of favourable response to CRT [10]. Furthermore, Barra et al. reported a low risk of malignant ventricular arrhythmias in a highly selected population with PICM, with a rate of sudden arrhythmic death of 6.2 per 1000 patient-years. In this cohort, the addition of defibrillator therapy was not associated with a significant survival benefit [11]. Kerley et al. also reported a low incidence of appropriate ICD therapies among patients with PICM after CRT upgrade, with most events occurring within the first year after the procedure [17]. Interestingly, predictors of LV reverse remodelling were less apparent. An absolute increase in LVEF of ≥10 percentage points occurred in 78% of patients, consistent with previous studies of CRT upgrade for PICM [7,11,12,17], but no significant predictors were identified in our cohort. Previous studies have associated QRS narrowing, nonischaemic cardiomyopathy, sinus rhythm, and a higher RV pacing burden with greater LVEF recovery after CRT upgrade [8,9,10,27,28,29]. Device-related infection requiring system extraction occurred in 5% of patients, a rate higher than that reported in the Danish registry [30]. This difference should be interpreted in the context of our small cohort and the higher-risk profile of patients undergoing CRT upgrade, who are generally older and have a substantial burden of comorbidities [31].

These findings may help estimate the likelihood of persistent LVEF ≤ 35% after CRT upgrade, which may inform consideration of ICD therapy for primary prevention. The choice between CRT-P and CRT-D in patients with PICM is challenging because the extent of LV recovery after resynchronization cannot be predicted reliably at the time of upgrade. Studies of selected patients with PICM have shown substantial recovery of LV systolic function, a low incidence of malignant ventricular arrhythmias, and no clear survival benefit from the addition of defibrillator therapy, suggesting that the incremental benefit of CRT-D may be limited in patients likely to recover. However, ICD therapy may remain beneficial in those with persistent severe LV dysfunction. Identifying these patients before CRT upgrade is therefore clinically relevant. In our cohort, patients with severe LV dysfunction and advanced LV dilatation were least likely to recover to an LVEF > 35%, suggesting that defibrillator therapy may warrant greater consideration in patients with this baseline LV characteristics. These findings should be considered hypothesis-generating and interpreted cautiously given the small sample size and observational study design, and they require prospective validation.

This study has several limitations. The small, single-center cohort and limited number of primary endpoint events restricted statistical power and multivariable analysis. Five patients did not complete the 12-month follow-up assessment and could therefore not be classified according to LV function at 12 months, potentially introducing survivorship bias. The LVEF and LVEDD thresholds were derived from the study cohort and require external validation. LVEF was visually estimated when biplane measurement was not feasible. The method of LVEF assessment was not systematically recorded, potentially affecting endpoint classification. Furthermore, LVEF assessment may have been influenced by measurement variability and pacing-related septal motion abnormalities. Although follow-up echocardiography was performed by a single experienced examiner, the absence of blinding to clinical information may have introduced observer bias. Physiologic pacing was not established at our center during the study period and therefore was not evaluated as an alternative pacing strategy. Finally, device selection was not randomized, and the study was not designed to compare CRT-P with CRT-D. The findings should therefore be considered hypothesis-generating.

## 5. Conclusions

In patients with pacing-induced cardiomyopathy, CRT upgrade was associated with substantial recovery of LV systolic function, with 82% of patients achieving an LVEF > 35% at 1 year. Severe baseline LV dysfunction (LVEF ≤ 25%) and advanced LV dilatation (LVEDD ≥ 65 mm) were associated with a lower likelihood of recovery above this threshold. These exploratory findings may aid risk stratification in patients undergoing CRT upgrade for PICM but should not directly guide CRT-P versus CRT-D selection. Further validation in larger prospective studies is warranted.

## Figures and Tables

**Table 1:** Baseline characteristics of patients with pacing-induced cardiomyopathy.

Characteristic	Pacing-Induced Cardiomyopathy (*n* = 60)
Age—years, median (IQR)	75 (72–80)
Female sex—*n* (%)	21 (35)
Body-mass index—median (IQR)	29 (26–32)
Ischemic heart disease—*n* (%)	24 (40)
Arterial hypertension—*n* (%)	51 (85)
Atrial fibrillation—*n* (%)	42 (70)
paroxysmal—*n* (%)	10 (24)
persistent—*n* (%)	20 (48)
permanent—*n* (%)	12 (29)
Valvular heart disease—*n* (%)	18 (30)
Chronic obstructive pulmonary disease—*n* (%)	6 (10)
Diabetes mellitus—*n* (%)	24 (40)
Chronic kidney disease stage ≥ 3—*n* (%)	37 (62)
Anemia—*n* (%)	23 (38)
**Device characteristics**	
Time to upgrade—months, median (IQR)	64 (25–99)
Ventricular pacing—%, median (IQR)	99 (94–100)
Pacemaker	55 (92)
ICD—*n* (%)	5 (8)
CRT-P—*n* (%)	43 (72)
CRT-D—*n* (%)	17 (28)
**Previous medication**	
Beta-blocker—*n* (%)	50 (83)
ACEi/ARB/ARNI—*n* (%)	44 (73)
MRA—*n* (%)	25 (42)
Loop diuretic—*n* (%)	35 (58)
Amiodarone—*n* (%)	21 (35)
Antiplatelet drug—*n* (%)	28 (47)
Anticoagulation—*n* (%)	39 (65)
DOACs—*n* (%)	11 (18)
Warfarin—*n* (%)	27 (45)
LMWH—*n* (%)	1 (2)

Abbreviations: IQR, interquartile range; ICD, implantable cardioverter-defibrillator; CRT-D, cardiac resynchronization therapy with defibrillator; CRT-P, cardiac resynchronization therapy with pacemaker; ACEi, angiotensin converting enzyme inhibitor; ARB, angiotensin receptor blocker; ARNI, angiotensin receptor and neprilysin; MRA, mineralocorticoid receptor antagonist; DOACs, direct oral anticoagulants; LMWH, low molecular weight heparin.

**Table 2:** Pacing-induced cardiomyopathy, clinical and echocardiographic response to CRT upgrade.

Parameter	Before Upgrade	12 Months After Upgrade	*p* Value
**Laboratory parameters**			
Creatinine—μmol/L, median (IQR)	124 (88–156)	120 (91–168)	**0.02**
NT-proBNP—ng/L, median (IQR)	2017 (978–4824)	1220 (642–2310)	**0.01**
Haemoglobin—g/L, median (IQR)	134 (124–146)	131 (118–140)	**0.02**
**Electrocardiographic parameters**			
QRS—ms, median (IQR)	187 (175–200)	140 (134–153)	**<0.01**
**Echocardiographic parameters**			
LVEF—%, median (IQR)	30 (24–35)	45 (37–52)	**<0.01**
LVEDD—mm, median (IQR)	58 (54–64)	56 (51–61)	**<0.01**
**Mitral regurgitation—*n* (%)**			0.07
None	0	2 (3)	
Mild	15 (25)	21 (35)	
Moderate	28 (47)	28 (47)	
Severe	17 (28)	9 (15)	
**Tricuspid regurgitation—*n* (%)**			0.62
None	2 (3)	1 (2)	
Mild	20 (33)	26 (43)	
Moderate	27 (45)	20 (33)	
Severe	11 (18)	13 (22)	
**Pulmonary hypertension—*n* (%)**			**<0.01**
None	16 (27)	40 (68)	
Mild	26 (43)	17 (29)	
Moderate	15 (25)	1 (2)	
Severe	3 (5)	1 (2)	
**NYHA classification—*n* (%)**			**<0.01**
NYHA I	0	4 (7)	
NYHA II	19 (32)	33 (57)	
NYHA III	41 (68)	21 (36)	
NYHA IV	0	0	

Abbreviations: CRT, cardiac resynchronization therapy; IQR, interquartile range; NT-proBNP, N-terminal pro–B-type natriuretic peptide; LVEF, left ventricular ejection fraction; LVEDD, left ventricular end-diastolic diameter; NYHA, New York Heart Association. Follow-up data on pulmonary hypertension and NYHA functional class were available in 59 and 58 patients, respectively.

**Table 3:** Patients with pacing-induced cardiomyopathy after CRT upgrade: comparison between patients with recovery of LVEF to >35% and those with persistent severe LV dysfunction (LVEF ≤ 35%). Univariable and multivariable analyses of predictors of persistent severe LV systolic dysfunction after CRT upgrade (LVEF ≤ 35%).

Variable	LVEF > 35% (*n* = 49)	LVEF ≤ 35% (*n* = 11)	*p*	Univariable OR (95% CI)	*p*	Multivariable OR (95% CI)	*p*
Age—years, median (IQR)	75 (72–80)	75 (73–80.5)	0.97	1.12 (0.46–2.73) †	0.80	—	**—**
Male sex—*n* (%)	31 (63)	8 (73)	0.73	1.55 (0.36–6.59)	0.55	—	**—**
Time to CRT upgrade—months, median (IQR)	59 (26–99)	89 (27–103)	0.61	1.03 (0.91–1.17) ‡	0.64	—	**—**
BMI—kg/m^2^, median (IQR)	29 (27–31)	27 (26–33)	0.57	0.78 (0.36–1.68) §	0.52	—	**—**
NT-proBNP—ng/L, median (IQR)	1991 (664–4373)	2313 (1816–7394)	0.08	1.02 (0.95–1.09) ¶	0.63	—	**—**
Baseline LVEF—%, median (IQR)	30 (25–35)	22 (20–25)	**<0.01**	0.46 (0.26–0.81) ‖	**0.01**	0.63 (0.32–1.24) ‖	0.18
Baseline LVEDD—mm, median (IQR)	58 (54–61)	65 (59–70)	**0.03**	3.73 (1.34–10.41) #	**0.01**	1.85 (0.53–6.39) #	0.33
Post-CRT QRS- ms, median (IQR)	140 (132–148)	154 (139–164)	**0.02**	1.81 (1.11–2.96) #	**0.02**	1.48 (0.83–2.65) #	0.18
QRS change—ms, median (IQR)	46 (36–56)	46 (34–55)	0.89	1.10 (0.71–1.72) #	0.68	—	**—**
LV lead position—*n* (%)			0.67	0.44 (0.05–3.93)	0.47	—	**—**
lateral	40 (82)	10 (91)					
posterior	9 (18)	1 (9)					
Ischemic heart disease—*n* (%)	17 (35)	7 (64)	0.10	3.29 (0.84–12.86)	**0.09**	1.60 (0.31–8.29)	0.57
Arterial hypertension—*n* (%)	44 (90)	7 (64)	**0.05**	0.20 (0.04–0.93)	**0.04**	0.25 (0.04–1.59)	0.14
Atrial fibrillation—*n* (%)	36 (74)	6 (55)	0.28	0.43 (0.11–1.66)	0.22	—	**—**
Valvular disease—*n* (%)	14 (29)	4 (36)	0.72	1.43 (0.36–5.66)	0.61	—	—
COPD—*n* (%)	5 (10)	1 (9)	1.00	0.88 (0.09–8.38)	0.91	—	—
Diabetes mellitus—*n* (%)	19 (39)	5 (46)	0.74	1.32 (0.35–4.92)	0.68	—	—
Anaemia—*n* (%)	19 (39)	4 (36)	1.00	0.90 (0.23–3.50)	0.88	—	—
Chronic kidney disease stage ≥ 3—*n* (%)	29 (59)	8 (73)	0.51	1.84 (0.43–7.79)	0.41	—	—
ACEi/ARB/ARNI—*n* (%)	36 (74)	8 (73)	1.00	0.96 (0.22–4.19)	0.96	—	—
MRA—*n* (%)	21 (43)	4 (36)	0.75	0.76 (0.20–2.95)	0.69	—	—
Beta-blocker—*n* (%)	41 (84)	9 (82)	1.00	0.88 (0.16–4.85)	0.88	—	—

Abbreviations: LVEF, left ventricular ejection fraction; IQR, interquartile range; BMI, body mass index; NT-proBNP, N-terminal pro–B-type natriuretic peptide; LVEDD, left ventricular end-diastolic diameter; CRT, cardiac resynchronization therapy; LV, left ventricular; COPD, chronic obstructive pulmonary disease; ACEi, angiotensin converting enzyme inhibitor; ARB, angiotensin receptor blocker; ARNI, angiotensin receptor and neprilysin; MRA, mineralocorticoid receptor antagonist. **Scaling of ORs:** † per 10-year increase; ‡ per 12-month increase; § per 5-kg/m^2^ increase; ¶ per 1000-ng/L increase; ‖ per 5-percentage-point increase; # per 10-unit increase.

**Table 4:** Prevalence of persistent severe left ventricular systolic dysfunction at 12 months after CRT upgrade according to baseline LVEF and LVEDD.

Baseline LV Characteristics	Persistent LVEF ≤ 35%
LVEF > 25% + LVEDD < 65 mm	0/27 (0%)
LVEF > 25% + LVEDD ≥ 65 mm	1/4 (25%)
LVEF ≤ 25% + LVEDD < 65 mm	5/18 (28%)
LVEF ≤ 25% + LVEDD ≥ 65 mm	5/11 (46%)

Abbreviations: LB, left ventricular; LVEF, left ventricular ejection fraction; LVEDD, left ventricular end-diastolic diameter.

## Data Availability

The data that support the findings are not openly available due to reasons of sensitivity but are available from the corresponding author upon reasonable request.

## Abbreviations

The following abbreviations are used in this manuscript:

RV	right ventricular
LV	left ventricular
PICM	pacing-induced cardiomyopathy
CRT	cardiac resynchronization therapy
LVEF	left ventricular ejection fraction
ICD	implantable cardioverter-defibrillator
IQR	interquartile range
NYHA	New York Heart Association
NT-proBNP	N-terminal pro–B-type natriuretic peptide
LVEDD	left ventricular end-diastolic diameter
CRT-P	cardiac resynchronization therapy with pacemaker
CRT-D	cardiac resynchronization therapy with defibrillator
LBBAP	left bundle branch area pacing
